# *Lactobacillus rhamnosus* Attenuates Cisplatin-Induced Intestinal Mucositis in Mice via Modulating the Gut Microbiota and Improving Intestinal Inflammation

**DOI:** 10.3390/pathogens12111340

**Published:** 2023-11-11

**Authors:** Duaa M. Alsholi, Ghazi Suleiman Yacoub, Ata Ur Rehman, Hidayat Ullah, Asif Iqbal Khan, Ting Deng, Nimra Zafar Siddiqui, Yamina Alioui, Nabeel Ahmed Farooqui, Maroua Elkharti, Yanxia Li, Liang Wang, Yi Xin

**Affiliations:** 1 Department of Biotechnology, College of Basic Medical Science, Dalian Medical University, Dalian 116044, China; doaa.alshouly@gmail.com (D.M.A.); ata_burraq@yahoo.com (A.U.R.); hidayat.khan89@yahoo.com (H.U.); asif.iqbal@duhs.edu.pk (A.I.K.); dtings123@163.com (T.D.); nimra.siddiqui12@gmail.com (N.Z.S.); yalioui@outlook.fr (Y.A.); nabeel.farooqui99@yahoo.com (N.A.F.); 2 Department of Dermatology, The First Affiliated Hospital of Dalian Medical University, 222 Zhongshan Lu, Dalian 116011, China; drghazi1988@gmail.com; 3 Department of Biochemistry and Molecular Biology, College of Basic Medical Science, Dalian Medical University, Dalian 116044, China; marouaelkharti@gmail.com; 4 Department of Respiratory and Critical Care Medicine, The First Affiliated Hospital of Dalian Medical University, Dalian 116011, China; 5 Stem Cell Clinical Research Center, National Joint Engineering Laboratory, Regenerative Medicine Center, The First Affiliated Hospital of Dalian Medical University, Dalian 116011, China

**Keywords:** *Lactobacillus rhamnosus*, probiotic, intestinal mucositis, cisplatin, proinflammatory cytokines, 16S rRNA fecal microbiome

## Abstract

*Lactobacillus rhamnosus* (LBS) is a well-documented probiotic strain in oncology and has a pivotal role in clinical applications. Here, we have investigated the protective effect of *Lactobacillus rhamnosus* on intestinal mucositis induced by cisplatin (CP) and explored the underlying mechanisms targeting inflammatory proteins, as well as the histological changes in the intestinal tissue of mice, in addition, the bacterial strains that may be related to the health-enhancing properties. BALB/c mice were pre-treated with or without LBS via oral gavage, followed by mucositis induction with cisplatin. Our results revealed that the LBS-treated groups significantly attenuated proinflammatory cytokine levels (IL-1β, IL-6, and TNF-α) compared to the CP group. Furthermore, LBS mitigated the damaged tight junction integrity caused by CP via up-regulating the levels of claudin, occludin, ZO-1, and mucin-2 protein (MUC-2). Finally, the 16S rRNA fecal microbiome genomic analysis showed that LBS administration enhanced the growth of beneficial bacteria, i.e., *Firmicutes and Lachnospiraceae,* while the relative abundance of the opportunistic bacteria *Bacteroides* and *Proteobacteria* decreased. Collectively, LBS was found to beneficially modulate microbial composition structure and functions and enrich the ecological diversity in the gut.

## 1. Introduction

The gut microbiome plays a pivotal role in maintaining the host’s health, potentially through regulating immune stability and protecting against gastrointestinal diseases [1,2]. A healthy gut microbiome is characterized by bacterial richness and ecological diversity [3], which enhance the integrity of the mucosal epithelium and protect against pathogenic microbes’ invasion [4].

Disturbed gut flora has been linked to various diseases, including inflammatory bowel disease (IBD) [5], depression [6], obesity [7], and type 2 diabetes [8]. Maintaining the balance in the richness and diversity of microbial communities appears to assist in the alleviation of various disorders.

Previous studies have shown that the biodiversity and richness of the gut microbiome can be changed significantly with chemotherapeutic drugs [9,10]. The structure and function of the intestinal barrier may be impaired through changes in host physiology, resulting in the pathogenesis of intestinal mucositis [11].

Intestinal mucositis represents one of the most frequent side effects in oncology patients undergoing chemotherapy. It is defined as the inflammation of the mucous membranes that line the digestive tract, resulting in structural, functional, and immunological abnormalities, also characterized by increased intestinal permeability, a reduction in mucin levels, and oxidative damage [12]. Cisplatin is one of the most potent platinum chemotherapeutic agents widely used as an effective therapy against various types of malignancies. For over four decades, CP has been commonly used for the management of cervical cancer, testicular cancer, and bladder cancer, with cure rates of over 90% [13,14,15]. CP is able to exert strong cytotoxic effects by blocking the DNA repair mechanism in tumor cells, preventing replication, and consequently triggering apoptosis [16]. However, the clinical application of CP is still limited, due to its toxicity. Moreover, the adverse effect of CP is well recognized with high-dose therapy, which includes hepatotoxicity, renal damage, nephrotoxicity, and damage to the intestinal epithelium [17]. Therefore, it is necessary to find an effective way to mitigate the intestinal barrier damage caused by cisplatin.

Research regarding cisplatin-induced alterations to the gut microbiome is yet to be conducted minutely. Furthermore, the restoration of the intestinal flora through probiotics has been a clinically promising therapeutic option for gut-associated disorders [18]. Intestinal injury is closely linked to inflammation, and treatment with probiotics has been found to have the potential to activate anti-inflammatory compounds, such as IL-10, and it has been reported to upregulate the expression of anti-inflammatory cytokines, including Interferon-γ, tumor necrosis factor-α (TNF-α), and inhibit the proinflammatory transcription factor NF-κB [19,20]. 

*Lactobacillus rhamnosus* (LBS) is a member of the lactic acid bacteria group (LAB), which is one of the most studied probiotic strains in oncology [21], as it exerts numerous beneficial effects [22,23]. In clinical application, LBS is a bacterium resident in the gut that can recover disturbed gut microbiota and demonstrate anti-inflammatory effects, as well as boost the immune system, which could accelerate the healing of intestinal epithelial homeostasis [24,25,26,27]. Also, it has been used in a wide range of other illnesses, reducing severe diarrhea, inflammatory bowel disease, ulcerative colitis, and yellow fever [28,29]. Previous studies have shown the efficacy of several LABs in regulating microbiome dysbiosis [30] and maintaining the intestinal epithelium integrity of tight junction proteins [31].

Here, we aim to investigate the protective effects and safety of probiotic *Lactobacillus rhamnosus* on cisplatin-induced intestinal injury and explore the underlying mechanisms targeting inflammatory proteins, as well as the histological changes in the intestinal tissue of BALB/c mice. In addition, we studied the bacterial strains that may be related to the health-enhancing properties via 16S rRNA sequencing.

## 2. Materials and Methods

### 2.1. Probiotic Strain

The *Lactobacillus rhamnosus* (LBS) strain was obtained from BeNa Culture Collection (Xinyang City, Henan province, China). The LBS were cultured in anaerobic conditions at 37 °C in De Mann Rogosa Sharpe solid medium (MRS) for 24 h. After that, a single colony was inoculated into MRS liquid medium and cultured overnight. Before administration to the mice, the LBS cultures were centrifuged at 6000× *g* at 4 °C for 10 min, then washed twice with physiological saline, and suspended to obtain the final concentration of 1 × 10^9^ CFU/mL.

### 2.2. Ethical Statement and Experimental Animals

Approval for animal ethics and experimental design was obtained from Dalian Medical University (Approval Code: 202110083). Forty-eight male BALB/c mice (5–6 weeks old, 18 ± 2 g body weight) were obtained from the Specific-Pathogen-Free Animal House Facility (SPF) of Dalian Medical University, and the committee guidelines from the National Institutes of Health for the care and handling of animals were followed. The mice were kept in sterilized cages at room temperature (22 ± 2 °C), with a humidity of 65% ± 5%, 12 h cycle of light and darkness, with free access to food and distilled water. 

### 2.3. Study Design

After one week of acclimation, the mice were randomly assigned to the following four groups (*n* = 12): normal control group (control), control + LBS, model group (CP), and model + probiotic group (CP.LBS). Saline (0.9% NaCl, wt/vol) was given to the control and LBS groups once daily via oral gavage. The mice in the CP and CP.LBS groups received an intraperitoneal injection of cisplatin at a dose of 6 mg/kg/d once daily for 3 days to cause intestinal mucositis (IM), while the mice in the LBS and CP.LBS groups received LBS orally from day 1 to day 7, in addition to LBS pre-treatment 1 week prior to IM induction. Also, from day 1 to day 3, saline was intraperitoneally administered to the mice in the control and LBS groups. The body weight and food and water consumption were documented every day until one day before the sacrifice of the animals by cervical dislocation, according to the Animal Care and Use Committee at Dalian Medical University.

Additionally, daily assessments of the mice’s health, including observations of their appetite, activity, fur, and feces, were carried out. The experimental design used in animal studies is described in the Supplementary Materials, which are based on the pre-experiments and previous research [32]. 

### 2.4. Measurement of Organ Indices

After the mice were sacrificed, the immunological organs, the thymus and spleen, were harvested and weighed immediately. The spleen and thymus indices were calculated using the following formula: spleen or thymus indices (mg/g) = weight of spleen or thymus (mg)/weight of mouse (g).

### 2.5. Stool Output and Diarrhea Assessment

After the induction of intestinal mucositis, stool samples of all of the mice were checked daily and the severity of diarrhea was assessed by using Bowen’s score system [33] to classify the stool consistency into the following four grades: 0. normal stool; 1. soft, slightly wet stool indicated mild diarrhea; 2. wet and unformed stool indicated moderate diarrhea; and 3. watery stool indicated severe diarrhea.

### 2.6. Pro-Inflammatory Cytokines Analysis

The whole blood was drawn through the eye orbit, and serum was obtained in a 1.5 mL tube and centrifuged at 2000× *g* for 10 min, then stored at −20 °C until further assessment. The cytokine concentrations (IL-1β, IL-6, and TNF-α) were measured using a mouse ELISA kit (Shanghai Longton Biotechnology Co., Ltd., Beijing, China), according to the manufacturer’s guidelines.

### 2.7. Real-Time Quantitative PCR (RT-qPCR)

Total RNA was extracted from colonic tissues with a TRIzol^®^ Reagent Kit (life technology, MA, USA). NanoDrop 2000 (Thermo Fisher Scientific, Wantham, MA, USA) was used to check the quantity of cDNA, and, using a commercial kit HiScript II Q RT SuperMix (Vazyme Biotech Co., Ltd., Nanjing, China), 2 μg of RNA was reverse transcribed to cDNA. A ChamQ SYBR qPCR MasterMix kit was used to measure the gene expression utilizing Bioer light gene 9600 analyzers (Hitech (Binjiang) District, Hangzhou, 310053, China). The following procedures were used for PCR cycling: 50 °C for 2 min, 95 °C for 10 min, and 40 cycles of 95 °C for 15 s and 60 °C for 1 min. The 2^−ΔΔCt^ equation was used to measure the relative gene expression level of the target genes using the instrument software gene 9660, as outlined by Livak et al. [34]. The kits for the primers used were bought from Invitrogen (Table 1). The GAPDH served as a control gene and the healthy control functioned as an endogenous calibrator [35].

### 2.8. Histological Examination

The colon and ileum tissues were excised and washed with cold PBS. About 5 µm of embedded tissue was sectioned using a microtome, and the tissue slices were fixed in 4% formalin for 24 h. The tissue was then deparaffinized in xylene twice for 10 min before being rehydrated using various ethanol gradients. Following that, the tissue was stained with hematoxylin and eosin (H&E). The microscopic examination for histological alteration was carried out with a light microscope (Leica Microsystems, Wetzlar, Germany).

### 2.9. Mucin Production and Goblet Cells

Immunohistochemistry (IHC) was used to examine the expression of mucin-2 (MUC-2) in the colon and ileum tissues. A 5-µm section of colon and ileum tissue was carefully cut, deparaffinized with xylene, and rehydrated in ethanol at various gradients, followed by incubation with 3% H_2_O_2_ for 10 min. For antigen retrieval, the tissue slide was warmed in antigen retrieval buffer (1 mM Na^+2^ EDTA, pH 8.0), then incubated overnight with primary antibodies, followed by incubation with horseradish peroxidase (HRP)-conjugated secondary antibody for 1 h. The slides were stained with 3,3-diaminobenzidine (DAB) as a substrate, and hematoxylin was used as a counterstain, according to the protocol of the immunohistochemical staining kits SP-KIT9720 (MXB Biotechnologies Biotechnology, Beijing, China). Afterward, the slides were fixed and visualized under a light microscope at 10× and 20× magnification, 100 µm scalebar.

### 2.10. Immunofluorescent Staining for Tight Junction Proteins

The levels of expression of claudin-1, occludin, and ZO-1 were assessed using immunofluorescent staining. A total of 5 µm of paraffin-embedded colonic and ileum tissue, which had been sliced and put onto a slide with a positive charge, was deparaffinized in xylene and then rehydrated in a succession of ethanol gradients. Citrate buffer was used to treat the tissue slices for antigen retrieval for 30 min at 100 watts in a microwave, followed by 1 h of cooling. The tissue slides were then included in blocking for 1 h with a 3% BSA solution and incubated at 4 °C overnight. DAPI was used to stain the nucleus and fluorescein (FITC)-conjugated secondary antibodies for 60 min. Images were taken using a confocal scanning microscope.

### 2.11. Gut Microbiome Genomic DNA Extraction and 16S rRNA Pyrosequencing

Total genomic DNA samples were extracted from fresh fecal samples using a Power Max (stool/soil) DNA isolation kit (MoBio Laboratories, Carlsbad, CA, USA) following the manufacturer’s instructions, then stored at −80 °C pending further analysis. The obtained genomic DNA was measured using a NanoDrop (Thermo Fisher Scientific, Waltham, MA, USA) to verify the DNA’s quantity and purity. Also, agarose gel electrophoresis was used to evaluate the DNA’s quality. The forward primer 515F (5′GTGCCAGCMGCCGCGGTAA-3′) and reverse primer 806R (5′-GGACTACHVGGGTWTCTAAT-3′) [36] were used to amplify the V4 region of the 16S rRNA gene from the whole genomic DNA extracts using the following procedure: the initial denaturation temperature was set at 98 °C for 30 s, followed by 25 cycles of denaturation at 98 °C for 15 s, annealing at 58 °C for 15 s, and extension at 72 °C for 15 s, with a final extension of 1 min at 72 °C. The sample sequencing was analyzed using the IllluminaNovoSeq6000 platform at GUHE Info Technology Co., Ltd. (Hangzhou, China). Microbial ecology software (QIIME software version 1.9.0) was used for sequence read processing and pipeline, as defined previously [37]. Also, the sequence read was analyzed, and the OUT with low quality was excluded through the following criteria [38,39]. The alpha diversity, richness, Shannon, Simpson, and evenness indices were assessed using the QIIME and R packages (v3.2.0). Additionally, the beta diversity was evaluated through many parameters, including UniFrac distance metrics, principal coordinate analysis (PCoA), principal component analysis (PCA), and non-metric multidimensional scaling (NMDS). Furthermore, the key biomarkers of the different groups were assessed using LEfSe (LDA) linear discriminant analysis effect size, which analyzes the predominance and differences in the species [37]. Each dataset’s taxonomic unit was determined using the Greengenes database.

### 2.12. Metagenomic Functional Analysis of the Microbiome Composition

The mouse gut microbiome is closely similar to its human homolog, with almost 95.2% of its KEGG orthologous groups shared, as reported by Xiao et al. [40]. KEGG Pathways are a set of pathway maps that illustrate the molecular correlation between the genetic information and metabolism. The 16S rRNA sequences were used to determine the functional diversity and abundance of the gut flora in the varied research groups. For the analysis of the abundance of the gene families with quantifiable uncertainty, PICRUSt was used to predict the important discoveries from the human microbiome project [41]. The resulting sequence file was further examined using the STAMP software package, version 2.1.3, as previously studied by Parks et al. [42]. Moreover, FAPROTAX [43] and BugBase [44] were used to analyze a diagram of ecologically related metabolites and functions in prokaryotic clades.

### 2.13. Statistical Analysis

The statistical data were examined using the software GraphPad Prism (7.00) (La Jolla, CA, USA). One-way analysis of variance (ANOVA) and Tukey’s multiple comparison tests were utilized to ascertain differences, and *p*-value *<* 0.05 was regarded as statistically significant. The LEfSe was studied using Kruskal–Wallis and Wilcoxon tests. The OUT and phenotype were statistically analyzed using the Mann–Whitney test.

## 3. Results

### 3.1. LBS Treatment Attenuates Body Weight Loss and Increases Food and Water Intake and Organ Index

**Figure 1:**
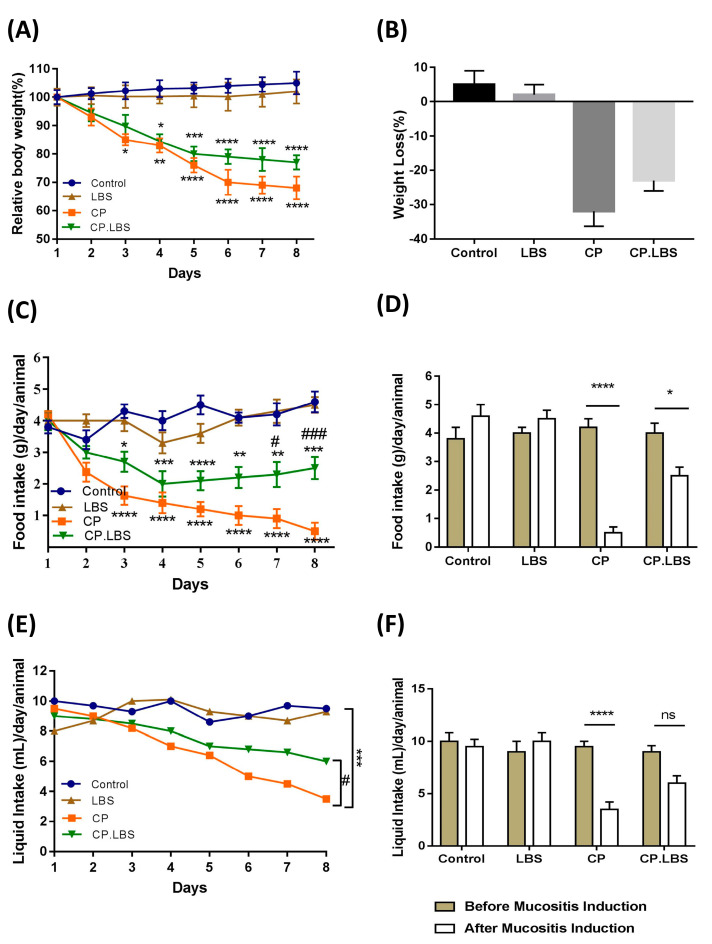
Effects of probiotic LBS on cisplatin−induced mucositis in mice: (**A**) Daily body weight variation. (**B**) Weight loss %. Change in the BW percentage % = BW on the specified day/the BW at day 0 × 100. (**C**) Daily food intake (g)/day/animal. (**D**) Food intake before and after mucositis induction. (**E**) Daily water variation. (**F**) Water intake. The results reflect the average of 3 separate trial ± SEM; * *p* < 0.05, ** *p* < 0.01, *** *p* < 0.001, and **** *p* < 0.0001 compared with the control group; # *p* < 0.05, and ### *p* < 0.001, compared to the model group; ns mean the difference not significant.

Body weight loss and anorexia (loss of appetite) are common symptoms that often occur during cisplatin treatment, and they are also basic signs of cisplatin toxicity. Before the induction of intestinal barrier damage, body weight, food, and water intake did not differ among the four groups. As expected, and observed in our experiments, the cisplatin-treated mice and cisplatin-combined-with-LBS-treated mice showed reductions of 31% ± 3.9 and 20% ± 2.5 approximately in body weight, respectively, compared with the control group (*p* < 0.0001), as shown in Figure 1A,B. Additionally, in the cisplatin-treated mice, there was a severe reduction in food intake by 90% (food intake: 0.5 ± 0.27 g and liquid intake: 3.5 ± 0.46 mL), compared with the control group *(p <* 0.0001). While co-administration of LBS with cisplatin ameliorates the mice’s appetite by 40%, (food intake: 2.52 ± 0.48 g and liquid intake: 5.98 ± 0.55 mL), it reduced significantly compared to the control group (*p* < 0.001) (Figure 1C–F). No significant change in body weight, food consumption, or water intake was observed in the LBS-alone group vs. the control group (food intake: 4.3 ± 0.55 g and liquid intake: 9.8 ± 0.60 mL vs. food intake: 4.5 ± 0.62 g and liquid intake: 9.5 ± 0.68 mL, respectively), as shown in Figure 1C–F.

Moreover, the general health state of the mice was monitored daily, and our observation indicates that the animals in the cisplatin group showed a severe reduction in activity, and the fur started to fall out after the cisplatin injections compared with the control group. In contrast, the mice in the CP.LBS group were more active, and their fur was in good condition compared to the model group.

**Figure 2:**
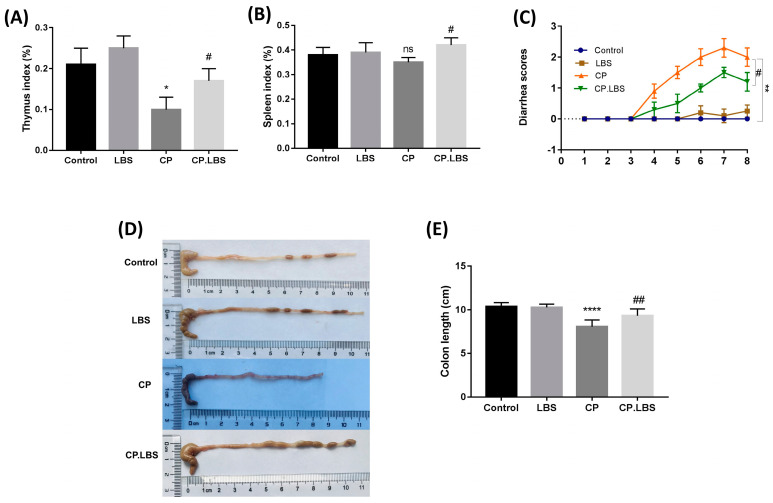
The LBS treatment resulted in improved organ indexing in cisplatin-induced mucositis in mice. (**A**) Thymus index (%). (**B**) Spleen index (%). (**C**) Diarrhea score. (**D**) The morphology of the mice colon. (**E**) The measurement of colon length. Data are presented as mean ± SEM; * *p* < 0.05, ** *p* < 0.01, **** *p* < 0.0001, and ns mean the difference statistically not significant compared with the control group; # *p* < 0.05, and ## *p* < 0.01, compared to cisplatin group. SEM, standard error of the mean.

Accordingly, the immunological organs’ (the thymus and the spleen) indices were reduced in the model group compared with the control group, while they improved significantly with the LBS treatment in the CP.LBS group compared to the model group, as shown in Figure 2A,B. The liver organ weight did not show a significant change in the model and treatment groups compared with the control group.

### 3.2. LBS Increases Stool Output and Reduces the Severity of Diarrhea

Fecal samples of the mice were monitored daily, and the results of all of the groups were compared. We noted a decrease in stool production in the cisplatin group compared with the control. Nevertheless, in the cisplatin + LBS group, the stool output remained lower than that in the control and LBS-alone groups. Indeed, no diarrhea was noted in the saline groups (LBS-alone and control group). On the contrary, in the cisplatin-treated mice, diarrhea started on day 5 after the cisplatin injections and developed into moderate diarrhea on day 6, 7 (*p* < 0.01), according to Bowen’s score system [33]. However, the diarrhea grade was significantly reduced in those mice treated with LBS in the CP.LBS group compared to the model group (*p* < 0.05) (Figure 2C).

Moreover, the colon length in the cisplatin-treated group was significantly shorter than that found among the other groups (CP 8.07 ± 0.27 vs. Con. 10.3 ± 0.2) (*p* < 0.0001), as shown in Figure 2D,E. Interestingly, the treatment with LBS had noticeably protective effects on the colon health, and it could improve the colon length shortening compared to the model group (CP.LBS 9.31 ± 0.3 vs. CP 8.07 ± 0.27) (*p* < 0.01) (Figure 2D,E).

### 3.3. LBS Attenuates Pro-Inflammatory Cytokine Levels in Cisplatin-Induced Intestinal Mucositis Mice Model

**Figure 3:**
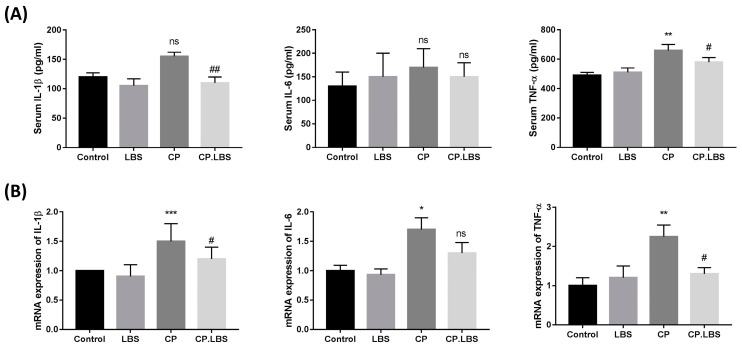
Measurement of pro-inflammatory cytokine (IL-1β, IL-6, and TNF-α). (**A**) Serum concentrations of cytokines measured with ELISA. (**B**) Relative expression of mRNA in colonic tissue. # *p* < 0.05, and ## *p* < 0.01, vs. CP group. * *p* < 0.05, ** *p* < 0.01, and *** *p* < 0.001, while ns is statistically not significant vs. control group. The results are presented as the mean ± SEM.

The mice in the CP group had significantly elevated serum levels of pro-inflammatory cytokine compared with those in the control group; however, the levels decreased significantly in the CP.LBS group compared with the model group, as shown in Figure 3A. A similar observation was noted in the colonic mRNA (Figure 3B).

### 3.4. Effects of LBS on Histopathological Examinations in the Intestinal Mucosal Layer 

**Figure 4:**
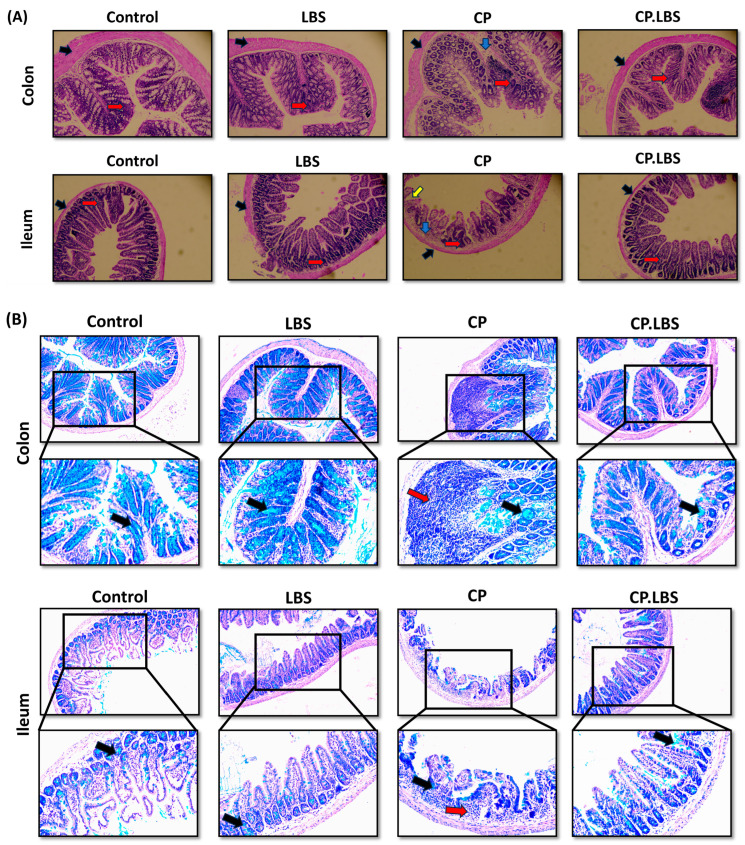
Effects of LBS on the histology of colon and ileum tissues after cisplatin treatment. (**A**) H&E images showing the healing effect of LBS on CP-induced IM. The blue arrow indicates inflammatory cells, the red arrow indicates epithelial and goblet cells, the black arrow indicates the epithelial surface, and the yellow arrow indicates the shortening of the villi, 10× magnification. (**B**) Alcian blue staining of colon and ileum tissues. The black arrow indicates the number of goblet cells and mucin production in each group, and the red arrow indicates inflammatory cell infiltration. Magnification: (upper 10×) and (lower 20×).

Hematoxylin and eosin (H&E) and alcian blue staining (AB) were examined in order to observe the histopathological changes, mucin expression, and goblet-cell production in the colon and ileum of the treated mice. The colon histology of the mice in the control group seemed to be normal and healthy, and the epithelium and goblet cells were both intact and uniformly arranged (Figure 4A,B). On the other hand, the cisplatin caused intestinal mucosal injury and elevated intestinal permeability in the CP group, which featured shortened intestinal villi, crypt depth disruption, and surface epithelial abrasion, combined with a reduction in the number of goblet cells (Figure 4A,B). However, the administration of probiotic LBS partly recovered these damages and the overall features of the ileum and colon and restored the loss of epithelial cells (Figure 4A,B).

**Figure 5:**
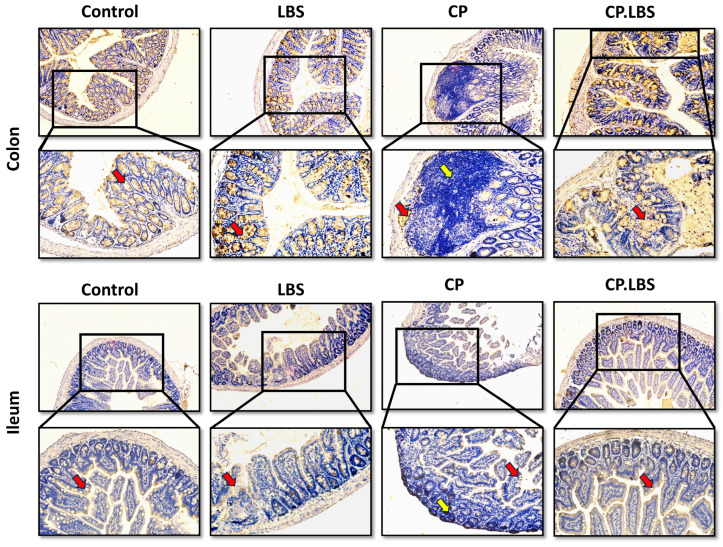
Mucin-2 immunohistochemistry staining in colon and ileum tissues of different groups. Mucin expression is indicated by a goldish color, as demonstrated with the red arrows, and inflammatory cells are shown in the model group, as demonstrated by the yellow arrows. Original magnification 10×, 20×, scale bar: 100 µm.

Furthermore, immunohistochemistry (IHC) was employed to assess the mucin-2 expression and the goblet-cell production in the colon and ileum tissues. Mucin-2 is considered to be the primary component of the intestinal mucosa released by the goblet cells [45]. In our study, a reduction was noted in the CP group. On the contrary, the probiotic LBS enhanced the mucus layer thickness and increased the expression of MUC-2 by recovering the epithelial cells and regenerating the number of goblet cells (Figure 5).

### 3.5. LBS Modulates the Tight Junction Protein Expression in the Colon and Ileum of CP-Induced IM Mice

**Figure 6:**
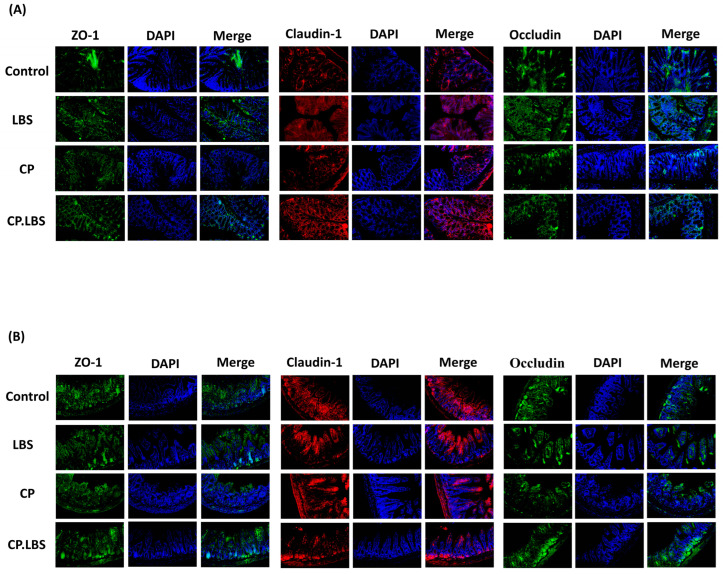
Immunofluorescent staining of tight junction proteins, including ZO-1, claudin-1, and occludin, in (**A**) colon tissue and (**B**) ileum tissue of different mice groups. Original magnification 20×, scale bar: 100 µm.

**Figure 7:**
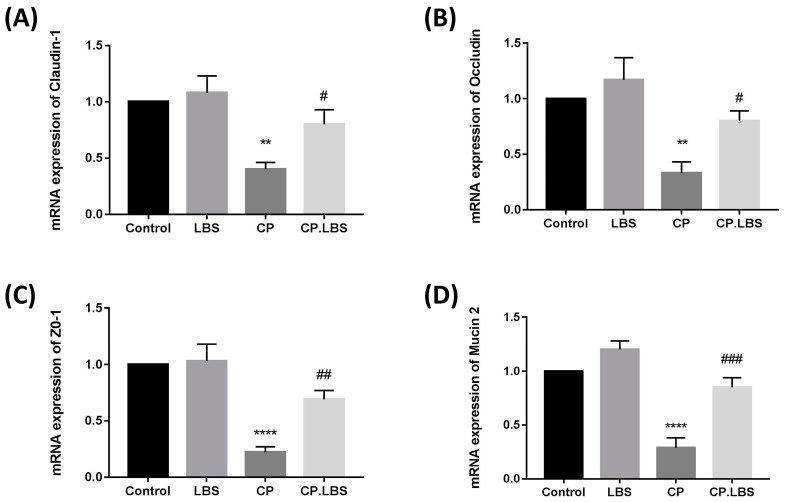
mRNA expressions in colonic tissue of (**A**) claudin-1. (**B**) occludin. (**C**) Zo-1. (**D**) mucin-2. # *p* < 0.05, ## *p* < 0.01, and ### *p* < 0.001, vs. CP group. ** *p* < 0.01, and **** *p* < 0.0001, vs. control group. The results are presented as the mean ± SEM.

Immunofluorescent staining was performed in order to explore the expression of ZO-1, claudin-1, and occludin in the colon and ileum tissues. The results demonstrated lower expressions of ZO-1, claudin-1, and occludin compared to those of the normal mice, while showing an enhancement in the relative expression levels of those tight junction proteins in the CP.LBS-treated mice compared to the model group (Figure 6). A similar observation was noted in the colonic mRNA expression for those tight junction proteins (Figure 7). These results prove that the LBS pre-treatment protects mice against cisplatin-induced intestinal mucosal damage.

### 3.6. LBS Treatment Modulates the Gut Microbiota Dysbiosis 

**Figure 8:**
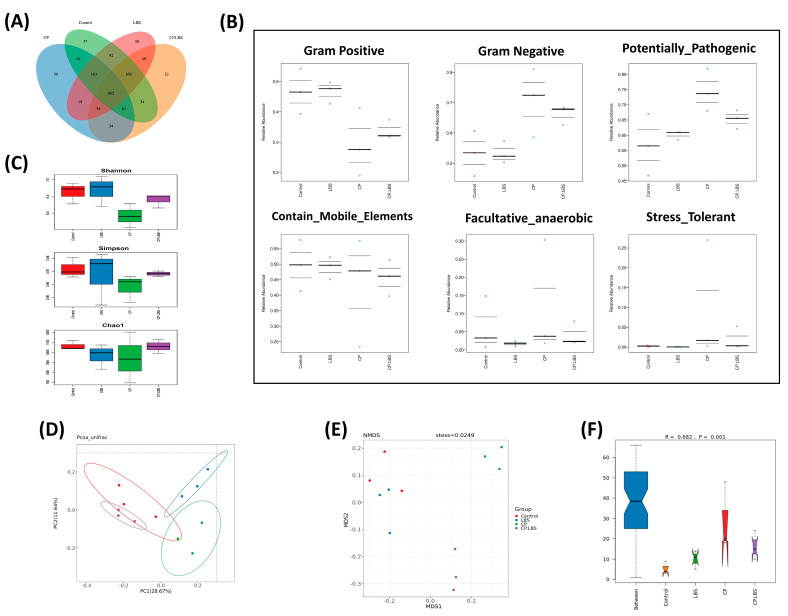
CP decreased the diversity and richness of the gut microbiome in the feces of the model mice groups. (**A**) Venn diagram demonstrates bacterial OTUs that are shared across treatment groups. (**B**) BugBase analysis identifies microbiome phenotypes that contain mobile elements, facultative anaerobic microorganisms, Gram-negative microorganisms, Gram-positive microorganisms, potential pathogens, and stress-tolerant microorganisms. (**C**) Alpha diversity indices, where Simpson measures the evenness in the community, while Shannon and Chao–1 indices measure bacterial community diversities and richness. (**D**–**F**) Gut microbiome beta diversity comparisons: (**D**) PCoA with Bray–Curtis dissimilarity, where different colored dots show mice receiving various treatments, and each colored dot indicates a particular animal; (**E**) Non–metric multidimensional scaling (NMDS); (**F**) The Anosim analysis, where the R value of >0 indicates that the difference between groups is greater than the difference within the group.

Cisplatin caused instability in the gut microbiome, which could be modulated by LBS administration in the cisplatin-treated mice. In the pyrosequencing targeting the V4 region of the 16S rRNA, there was a 97 percent similarity level between the operational taxonomic units (OTUs) within the range of 600–1000. According to the Venn diagram, 802 OTUs were shared between the control and the experimental groups. In addition, we observed the following differences among the groups: the control group, LBS group, and CP.LBS group noted a significant elevation in OTUs, while decreased OTUs were found in the CP-treated group (Figure 8A).

The BugBase microbial phenotypes results indicate elevated facultative anaerobic relative abundance and Gram-negative bacteria in the CP group, while Gram-positive bacteria relative abundance increased in the probiotic-supplementation group. Notably, LBS reduced the potential pathogens’ relative abundance and the stress-tolerant microbes that increased in the CP group, as shown in Figure 8B.

Also, a higher index level of alpha diversity has indicated a more diverse bacterial community. We found that CP decreased the α-diversity of the intestinal bacteria, which was demonstrated by the decline in the Chao-1, Simpson, and Shannon indices (Figure 8C). However, the reduction in the microbial community diversity and richness indices was reversed with the LBS administration. These findings suggest that LBS enhances ecological diversity in the gut.

Moreover, in order to demonstrate the gut microbial structures and to reveal the similarity or dissimilarity of the samples in the species compositions, the beta diversity pattern was analyzed by using principal component analysis PCoA (weighted UniFrac analysis) (Figure 8D) and non-metric multidimensional scaling (NMDS) (Figure 8E). The Anosim analysis is shown in Figure 8F. According to Bray–Curtis algorithm, the R value was >0, indicating that the difference between the groups is greater than the difference within the group. Our results have exhibited that the control, LBS, and CP.LBS groups were closer to each other than to the CP-alone group, suggesting that cisplatin induces variation in the gut flora, while the LBS treatment was more similar to the control group.

**Figure 9:**
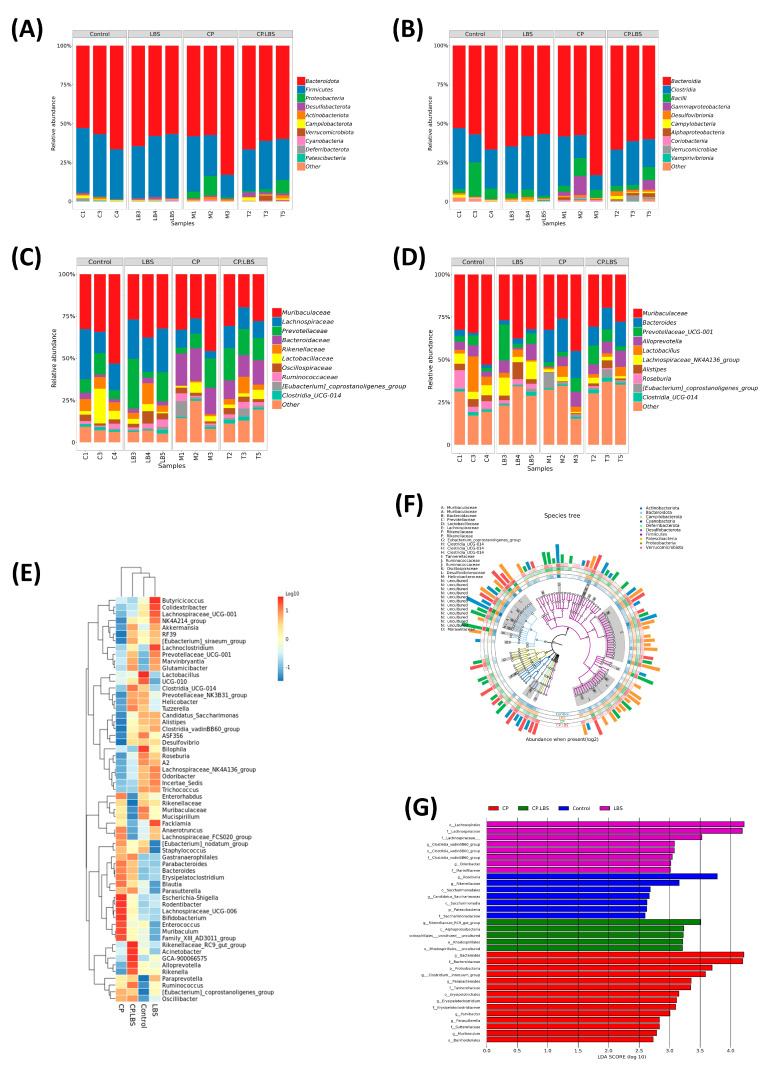
Impact of LBS on modifications to the colon microbiome. (**A**–**D**) Phylum, class, family, and genus level comparison among different groups. (**E**) Heatmap analysis of highly characterized bacterial level clusters of the gut microbiome into hierarchical clusters. (**F**) GraPhlAn circular image of the phylogenic tree from the extensive collections of the 16S rRNA metadata groups. (**G**) LEfSe analysis bars showing the taxonomic biomarker from phylum to genus between the experimental groups.

The taxonomic classification level (phylum, class, family, and genus) was identified for the intestinal flora in order to study the specific changes caused by CP and LBS in all of the treatment groups. In our results, the bacterial composition showed variation at all levels in the CP group, as compared to the control group. Furthermore, the dominant three bacterial phyla in the mouse gut composition are *Bacteroidetes*, *Firmicutes*, and *Proteobacteria*. In the cisplatin-treated mice, the relative abundances at the phylum level of the three dominant bacteria, respectively, was *Bacteroidetes* > *Firmicutes* > *Proteobacteria*. In the model group, the relative abundances of *Firmicutes* significantly decreased, while *Bacteroidetes* and *Proteobacteria* increased, compared with the other three groups (Figure 9A). However, the CP.LBS-treated mice mitigated the CP-induced phylum-level alteration. Moreover, there was no significant change in the phylum levels between the control group and the LBS groups shown, as in Table 2.

At the class level (Figure 9B), the cisplatin group displayed a higher relative abundance of *Bacteroides* and *Gammaproteobacteria*, but a lower relative abundance of *Clostridia* and *Bacilli* compared to the other three groups. At the family level (Figure 9C), the results reveal that the changes in the abundances between the four groups, *Lactobacillaceae*, *Lachnospiraceae*, and *Rikenellaceae,* were less abundant in the CP group as compared to the control, LBS, and CP.LBS groups, and a greater abundance of *Bacteroidaceae* level.

Interestingly, at the genus level, the cisplatin-injected mice demonstrated a decline in the abundances of *Lachnospiraceae_NK4A136*_group, *Lactobacillus*, *Alistipes*, and *Rosburia,* and an enriched abundance of *Bacteroides*; moreover, these changes were ameliorated with LBS supplementation (Figure 9D).

Inclusively, our findings have revealed that, at the taxonomic levels, the bacterial community was altered by the cisplatin drug in the CP mice, while the LBS treatment partially restored the gut dysbiosis. Heatmaps were used to assess the relative abundances of the bacterial genera (Figure 9E). Additionally, the top 10 species were selected to draw a phylogenetic tree using GraPhlAn (Figure 9F), and we investigated the taxonomic biomarkers using linear discriminative analysis effect size (LEfSe) (Figure 9G). Potentially, the enteropathogenic bacteria phylum *Proteobacteria*, family *Bacteroidaceae*, and genus *Bacteroids* were the predominant biomarkers in the cisplatin group, while genus *Rikenellaceae* was the predominant biomarker in the CP.LBS group. The beneficial bacteria family *Lachnospiraceae* were predominant biomarkers in the LBS-treated group. The genus *Rosburia* and *Rikenellaceae* were highlighted in the control group.

### 3.7. LBS Effect on the Gut Metabolic Functional Profile

**Figure 10:**
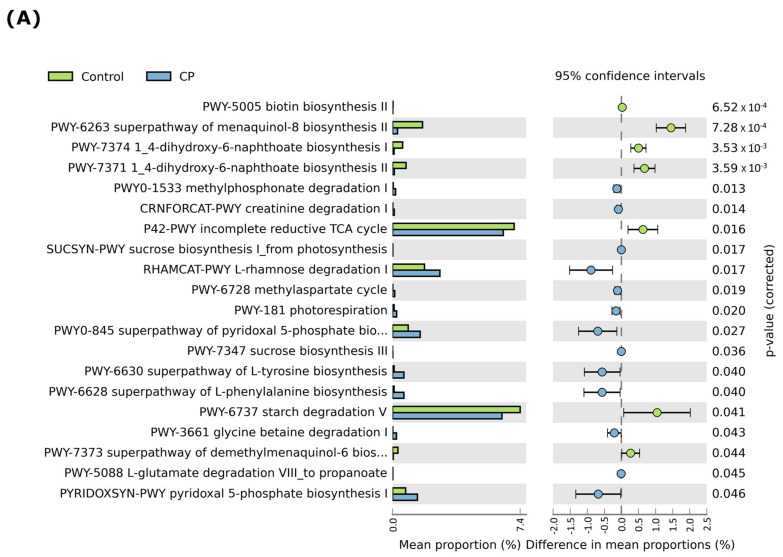

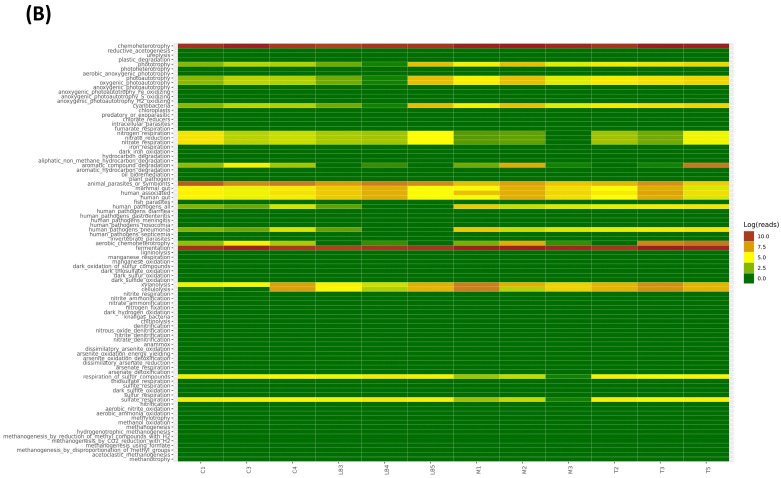
Analysis of the functional metabolic pathways in the control, model-CP, and treated groups. (**A**) KEGG STAMP analysis. (**B**) Heatmap analysis by the FABROTAX database, demonstrated to identify metabolic functions and other ecologically significant activities in the groups.

The gut metagenome of the microbial communities’ analysis from 16S rRNA using STAMP (version 2.1.3) and KEGG pathways showed differences between the CP-treated group and the control group. Interestingly, the most enriched metabolic pathways among these were as follows: sulfate and nitrogen respiration, amino acid biosynthesis, metabolism, starch degradation, creatinine degradation, L-rhamnose degradation, glycolysis, citrate cycle (TCA), energy production, photorespiration, biotin biosynthesis, sucrose biosynthesis, L-tyrosine, L-phenylalanine biosynthesis, and pyridoxal 5-phosphate biosynthesis. Together, the KEGG pathways were altered in the different groups, proving that the LBS treatment can boost immunity by modulating the gut microbiota’s metabolism (Figure 10A,B).

## 4. Discussion

In the current study, we used a BALB/c mouse model to investigate the protective effect of commensal probiotic LBS on gut microbiota dysfunction in cisplatin-induced intestinal mucositis. Cachexia (involuntary loss of weight > 5%), a typical side effect of chemotherapy that decreases survival in oncology patients, is a significant health issue that affects cancer patients. Weight loss in cancer patients also causes the detrimental effect of malnutrition as a result of anorexia, which can result in infection and life-threatening conditions. Our study is in accordance with that of Alhadeff et al., who reported that cisplatin caused dramatic weight loss and severe anorexia [46]. Additionally, pre-treatment with LBS attenuates the loss of weight, improves food intake, and increases stool output. Interestingly, LBS could improve gastrointestinal function and promote intestinal health.

Moreover, the thymus and the spleen are the main components of the immune system in the body and play a vital role in nonspecific immunity; in addition, they are considered the site of the proliferation of immunological cells [47]. Consequently, the immune organ index is usually used to indicate the growth of the immune organs and assess the role of probiotics in immunoregulation [48,49]. *Lactobacillus* has been reported previously to have an impact on activating nonspecific immunity [50,51]. Meng et al. and Li et al. have reported that some types of *lactobacilli* significantly enhanced the immune organ index [52,53], and that is consistent with our study, which has revealed that the thymus and spleen indices in three groups, including control mice and those with LBS treatment, were greater than those in the CP group. These results have indicated that LBS could resist the influence of cisplatin in the immunosuppression of the immune organs.

Given that, the intestinal epithelial cells (IECs) play an essential role in regulating intestinal homeostasis and are considered part of the immune system, as they take part in the transmission of the signal to the intestine through the secretion of cytokines and oxidative stress mediators [54]. Cytokines are low-molecular-weight glycoproteins that are produced by various cells in the body and have a crucial role in the progression of the immune response and the pathogenesis of inflammatory disease [55]. The effect of LBS on the production of IL-2, IL-6, and IFN-γ was determined and suggested to play a significant role in intestinal mucositis [56]. Previous studies have reported that pro-inflammatory cytokines are significantly increased in the large intestine of rats following post-treatment with chemotherapy [57]. Indeed, IL-1β, IL-6, and TNF-α are significantly upregulated in the serum and/or colon tissue at the mRNA levels following the administration of cisplatin and decreased levels in mice receiving CP.LBS. Our results are in agreement with the previous studies that endeavored to target the pro-inflammatory cytokines as a precautionary measure for intestinal barrier damage [58,59,60,61]. This has indicated that LBS could reduce the inflammation and, therefore, enhance those pro-inflammatory mediators involved in the progression of mucositis.

Also, tight junction proteins (TJs) offer a physical barrier to the intestine that contributes to maintaining intestinal barrier function, enhancing GI permeability, and maintaining the intestinal mucosal barrier [62]. TJs are composed of two protein categories, integral transmembrane proteins that form a connection between the neighboring cell membranes, like claudin and occludin, and peripheral membranes, like ZO-1, which connects claudin and occludin, which may serve to keep the tight junctions intact [63]. Therefore, TJ integrity is dramatically maintained by the strong bond between the integral transmembrane and the peripheral membrane protein, in addition to the arrangement of the actin cytoskeleton.

Moreover, decreases in the TJ levels always revealed an elevated permeability of the intestinal epithelial cell barrier [62,63,64]. Leocádio et al. and Beutheu Youmba showed that chemotherapeutic agents caused an elevation in intestinal permeability that led to the damage of the epithelial barrier through lowering the protein expression level of the TJs [65,66]. The maintenance of the integrity of the TJs suggests an important strategy to prevent and/or treat the pathogenesis of illness and intestinal damage. However, the mucin secreted by the goblet cells in the intestine is also important for creating the intestinal barrier [67]. The intestinal mucus could protect the intestinal epithelium against microbes by removing harmful bacteria [68].

In the CP model group, the staining results have indicated intestinal and mucosal barrier alteration and a reduction in the tight junction proteins. This led to epithelial cell damage, inflammatory cell infiltration, and a decrease in the availability of goblet cells. The LBS restores the damaged TJs’ integrity caused by CP by restoring the goblet cells and improving the tight junction stability. In keeping with these observations, an up-regulated level of occludin has a role in further improving the TJ integrity and preventing disorders of the TJs [69].

This leads us to explore further whether there is a difference in the gut microbiome structure or composition involved in the development of mucositis. The protective effect of LBS and the restoration of microbiota was examined using microbiota 16S rRNA pyrosequencing. The intestine plays a significant role in homeostasis. The microbiota mainly interacts to enhance the barrier integrity. However, diseases associated with metabolic disorders and immune suppression can lead to an imbalance in the microbial ecology and reduce the diversity and richness of the gut microbiome [70].

About 5 to 7 of the 52 identified bacterial phyla on earth are known to live in the mammalian gut. *Bacteroidetes* and *Firmicutes* typically are the most common and have the highest relative abundance in the gut, whereas the phyla of *Proteobacteria*, *Actinobacteria*, and *Verrucomicrobia* are found less frequently [71]. Moreover, our experimental groups showed variations in the intestinal microbiota. Indeed, the model group showed an increase in the abundance of pathogenic bacteria *Bacteroidota* and *Proteobacteria* (mucosa-associated inflammation-promoting bacteria) at the phylum level and a decrease in beneficial bacteria *Firmicutes*, when compared with the other three groups; moreover, the LBS-treated groups showed a reversed alteration induced by the cisplatin.

However, in a healthy intestine, the gut flora normally has a minor abundance of phylum *Proteobacteria*, and the increased appearance of these bacteria in the gut refers to an imbalanced microbial community (dysbiosis) and has been reported to be elevated in chemotherapy-induced mucositis [70,72,73].

Obligate anaerobic bacteria are responsible for converting many fermentation products into short-chain fatty acids [74,75]. The gut microbiota of a healthy colon is dominated by obligate anaerobes, whereas dysbiosis is often characterized by an increase in facultative anaerobic bacteria. Thus, in the large intestine, the dominance of obligate anaerobic bacteria maintains the gut stability via the production of metabolites. Indeed, our results support the hypothesis that an imbalanced gut microbial community is characterized by the enrichment of those facultative anaerobic bacteria. To sum up, we have suggested that the intestinal inflammatory response caused by cisplatin is associated with the overgrowth facultative anaerobic bacteria, such as *Proteobacteria* and *Actinobacteria,* while the CP.LBS group showed less abundance of those bacteria and was more similar to the control group.

Given that a leaky gut is demonstrated by a rise in Gram-negative bacteria, *Proteobacteria* is considered a Gram-negative bacterium, and their cell wall is mainly composed of lipopolysaccharide. The risk of disease is associated with the secretion of LPS, which is positively correlated and triggers inflammation [76]. Therefore, it may be hypothesized that the microbes that are identified as having potential for overgrowth in the CP group may relate to the inflammatory response in the intestine and may induce mucositis.

Undoubtedly, at the genus level, the beneficial bacteria, including *Lachnospiraceae*, *Lactobacillus*, *Alistipes*, and *Roseburia*, were observed in the LBS groups and decreased dramatically in the cisplatin group. Those bacteria play a key role in the metabolism of undigested carbohydrates [77] and produce butyrate and short-chain fatty acids (SCFAs) through hydrolyzing the starch and sugars that contribute to increasing the energy extracted from the diet [78,79,80]. Furthermore, clinical studies have shown that *Lachnospiraceae* is vital in attenuating intestinal inflammation and repairing intestinal mucosal damage, serving as protective intestinal commensal bacteria [71]. Moreover, previous studies have reported that *Lactobacillus* species upregulate the mucin content that is inhibited by cisplatin chemotherapy. It is suggested that the dysfunction of the mucus barrier may contribute to cisplatin-induced mucositis [81].

We have further studied the metabolome functions from 16S rRNA data by using bioinformatics tools. Dysbiosis has side effects on the metabolic and functional pathways and has an impact on the physiological processes of the organism, including the host immune system and nutrient biosynthesis, as studied in the KEGG orthologous analysis. Ultimately, in our study, a 16S rRNA sequencing analysis and a STAMP analysis were used to investigate the metabolome of the mouse gut microbiome. We observed that LBS may improve the method of utilizing energy, carbohydrate metabolism, and nutrient absorption, implying that LBS may act as an immunoprotective agent. We hope that our findings will lead to the development of novel, probiotic-based therapies for intestinal mucositis associated with antineoplastic therapy. Thus, further experiments should be explored on humans.

## 5. Conclusions

In summary, our study unveils the therapeutic potential of *Lactobacillus rhamnosus* in modulating the intestinal inflammation in CP-induced mucosal barrier damage in mice. LBS demonstrates its beneficial effects by improving several factors, including body weight loss, food and water consumption, stool production, diarrhea severity, colon length shortening, goblet cell regaining, and increasing mucin production. As a result, the pro-inflammatory cytokines were reduced, and the tight junction proteins were up-regulated. In addition, LBS enhances the microbiome diversity positively and increases the bacteria *Lachnospiraceae* and *Lactobacillus*. In turn, this regulates the gut microbiota imbalances caused by cisplatin and provides great potential in mitigating intestinal mucositis as a dietary agent.

## Figures and Tables

**Table 1:** List of primers used to assess mRNA gene expression using real-time PCR.

Gene	Forward Primer	Reverse Primer
GAPDH	AACGACCCCTTCATTGAC	CCACGACATACTCAGCAC
IL-1β	CTCCATGAGCTTTGTACAAGG	TGCTGATGTACCAGTTGGGG
IL-6	TGTGCAATGGCAATTCTGAT	GGTACTCCAGAAGACCAGAGGA
TNF-α	CATCTTCTCAAAATTCGAGTGACA	TGGGAGTAGACAAGGTACAACCC
Claudin1	ATCGCAATCTTTGTGTCCACCATT	ATTCTGTTTCCATACCATGCTGTG
Occludin	ACTCCTCCAATGGACAAGTG	CCCCACCTGTCGTGTAGTCT
ZO-1	AACCCGAAACTGATGCTATGGA	GCGGCCTTGGAATGTATGTG
Mucin-2	GATGGCACCTACCTCGTTGT	GTCCTGGCACTTGTTGGAAT

**Table 2:** The percentages of bacterial phylum in different treatment groups.

	Control	LBS	CP	CP.LBS
*Bacteroidota*	59.33%	59.81%	66.22%	62.64%
*Firmicutes*	37.78%	37.81%	25.46%	29.98%
*Proteobacteria*	0.24%	0.07%	5.86%	3.20%
*Desulfobacterota*	0.57%	0.99%	0.65%	1.90%
*Actinobacteriota*	0.69%	0.36%	1.21%	0.78%
*Campilobacterota*	0.96%	0.35%	0.19%	1.14%
*Cyanobacteria*	0.03%	0.28%	0.37%	0.37%
*Deferribacterota*	0.67%	0.15%	0.06%	0.04%
*Patescibacteria*	0.30%	0.25%	0.03%	0.09%
*Verrucomicrobiota*	0%	0%	0%	0%

## Data Availability

The original data for this work are available upon email request to the corresponding author.

## Supplementary Materials

The following supporting information can be downloaded at: https://www.mdpi.com/article/10.3390/pathogens12111340/s1, Figure S1: Animal experimental design.
