# Supplementary material for: Lactobacillus rhamnosus Attenuates Cisplatin-Induced Intestinal Mucositis in Mice via Modulating the Gut Microbiota and Improving Intestinal Inflammation
Source: Pathogens. 2023 Nov 11;12(11):1340. doi: 10.3390/pathogens12111340 (PMC10674506; doi:10.3390/pathogens12111340)
Supplement: Supplementary file 1 [file pathogens-12-01340-s001.zip › pathogens-2625501-supplementary.pdf]

Supplementary Materials:

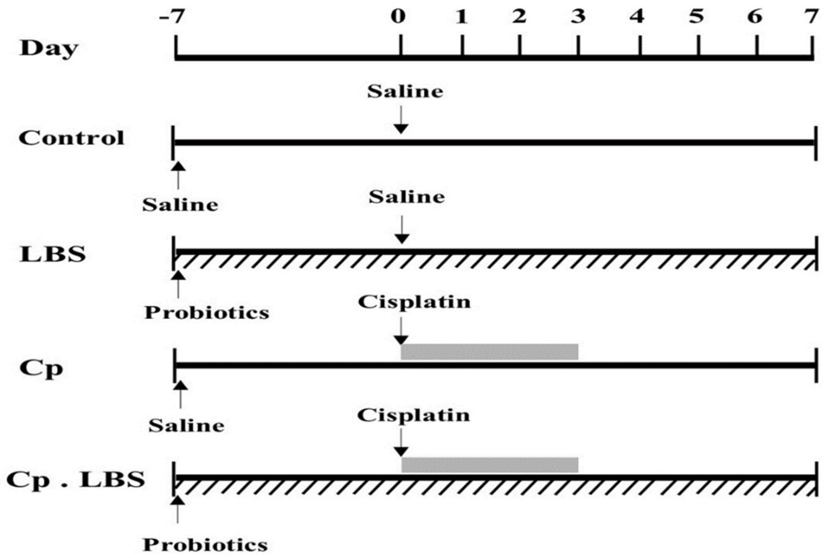

Figure S1: Animal experimental design. Animals were divided into four groups: control, LBS, CP, and CP.LBS. *Lactobacillus rhamnosus* LBS were given using oral gavage to LBS and CP.LBS group, Intestinal mucositis was induced in CP and CP.LBS group by intraperitoneal injection of cisplatin.
